# A Data Driven Approach to Achieving High Value Healthcare

**DOI:** 10.5334/egems.241

**Published:** 2017-12-15

**Authors:** Lucy A. Savitz, Lisa T. Weiss

**Affiliations:** 1 Kaiser Permanente Center for Health Research, US; 2 High Value Healthcare Collaborative, US

**Keywords:** high-value care, collaboration, data sharing, evidence generation

## Abstract

The purpose of this special issue is to disseminate learning from the High Value Healthcare Collaborative (HVHC). The HVHC is a voluntary, member-led organization based on trusted, working relationships among delivery system leaders. HVHC’s mission is to be a provider-based learning health system committed to improving healthcare value through data, evidence, and collaboration. We begin by describing the organization and structure of HVHC in order to lay the context for a series of papers that feature work from this learning health system. HVHC was awarded a grant from the John and Laura Arnold Foundation to develop a generalizable model for dissemination and implementation. Implementation of the 3-hour sepsis bundle was used as a prototypic, complex intervention with an in-depth mixed methods evaluation across 16 member sites. The first four articles in this issue describe, in detail, various data and methodological challenges encountered together with strategies for overcoming these (see Knowlton et al., von Recklinghausen et al., Welch et al., and Taenzer et al.). Next, we illustrate how the Data Trust can support emerging questions relevant to member organizations. The paper by Albritton et al., explores the impact of observation stays on readmission rates. Knighton et al., explore the use of an area-based measure for health literacy to assess risk in disadvantaged populations. Two final papers illustrate the importance of fundamental data sources needed to support advanced data science.

## Background and History

The purpose of this special issue is to disseminate learning from the High Value Healthcare Collaborative or HVHC. We begin by describing the organization and structure of HVHC in order to lay the context for a series of papers that feature work from this learning health system.

The HVHC is a voluntary, member-led organization based on trusted, working relationships among delivery system leaders. HVHC’s mission is to be a provider-based learning health system committed to improving healthcare value through data, evidence, and collaboration. To accomplish this, they:

- Measure, innovate, test, and continuously improve value-based care;
- Rapidly disseminate and facilitate adoption of proven high value care models across HVHC members and beyond; and
- Advocate for policy and payment models that support sustainable high value healthcare.

HVHC began with five founding members, grew at one point to 19 members, and has settled at the time of this writing on 13 member delivery systems (see **Box 1**). This fluctuation in membership is consistent with a living systems theory of organizational behavior whereby the collaborative can be thought of as a living, social organism—not static—that expands and contracts based on the changing needs and priorities of the group and its members over its lifespan [2,6,7]. As a collaborative that represents a cross-section of the healthcare industry (i.e., geography, teaching status, size, religious affiliation, and payer mix that includes both critical access and safety net hospitals), it is important to understand the history behind formalization of the HVHC, which was founded as a strategy aimed at leading the way in adapting to a complex, changing environment.

**Box 1: HVHC Membership, June 2017.**

- Baylor Scott & White Health
- Beth Israel Deaconess Medical Center
- Dartmouth-Hitchcock Medical Center
- Denver Health
- Hawaii Pacific Health
- Intermountain Healthcare
- The Mayo Clinic
- Northwell Health
- Providence Health & Services
- Sentara Health
- The Dartmouth Institute (TDI)
- UC San Diego
- Virginia Mason Medical Center

The Mayo Clinic and Intermountain Healthcare had been identified in the Dartmouth Atlas as low-cost, high-quality providers and were brought together informally by Dr. Jack Wennberg and others from The Dartmouth Institute (TDI) for over a decade. In the Fall of 2009 with Washington policy makers focused on healthcare reform, these groups—along with Dartmouth-Hitchcock Medical Center, Denver Health, and others—decided to expand and formalize a collaborative to establish a collective voice for delivery systems interested in leading the way on healthcare transformation and payment reform. Creating a collective voice and safe environment for shared learning was not a new idea. Organizational scientists have talked about the promising notion of strategic alliances as a vehicle for health services innovation [1]. The press dubbed HVHC as the “dream team” for these purposes [7] as member organizations executed a memorandum of understanding.

HVHC founding members were committed to using a data-driven approach in shared learning. As such, they decided to use a centralized model for data sharing, creating a Data Trust that was and continues to be managed by a centralized Program Management Office (PMO) housed at TDI, the data convener and coordinating center for HVHC (see Data Trust section below). Issues around data privacy, access, and transparency together with policies and procedures for governance led to hiring independent counsel to execute a Master Collaborative Agreement or MCA (see MCA section below). This data-driven approach is the core of HVHC discovery process, highlighting areas of high variation and high cost among members to target areas for shared learning and monitor improvements over time (see Discovery Process below).

*Great groups* have to invent a leadership style and structure that suits them [8]. At its inception, HVHC was led by a small group of founding members with support from the PMO. The founding members constituted the Steering Committee, holding decision-making authority for the Collaborative; and with continued membership growth, senior leaders from newly added member organizations served on an Executive Committee to represent the collective voice of the members and make HVHC decisions on behalf of their individual institutions. HVHC is currently evolving its governance structure as a member-led organization to be more inclusive in representation of all member organizations in response to recommendations from a recent Task Force review (see Governance Structure below).

As a member-led organization, alignment of member delivery systems’ strategic priorities with the work of the HVHC is an important success factor. Other key factors contributing to our success have been leveraging pre-existing relationships, starting small then expanding, creating a safe environment for data-driven shared learning, establishing and evolving a governance structure that meets HVHC’s changing needs, and being responsive by demonstrating value to members.

## HVHC Master Collaboration Agreement

HVHC is not a legal entity but rather a contractual agreement among participating Members under a centralized Master Collaboration Agreement (MCA). All HVHC Members execute and abide by the provisions of this agreement in the following areas: Members and Membership; Governance of the Collaborative; Data Sharing Requirements; Use of Data Submitted by Members; Collaborative Work Process and Funding; Publications; Intellectual Property; TDI Services; Liability, Insurance and Warranties; Confidentiality, Term and Termination. More on the MCA can be found in an overview article by McGraw and Letter [3].

## HVHC Governance

**Figure 1:**
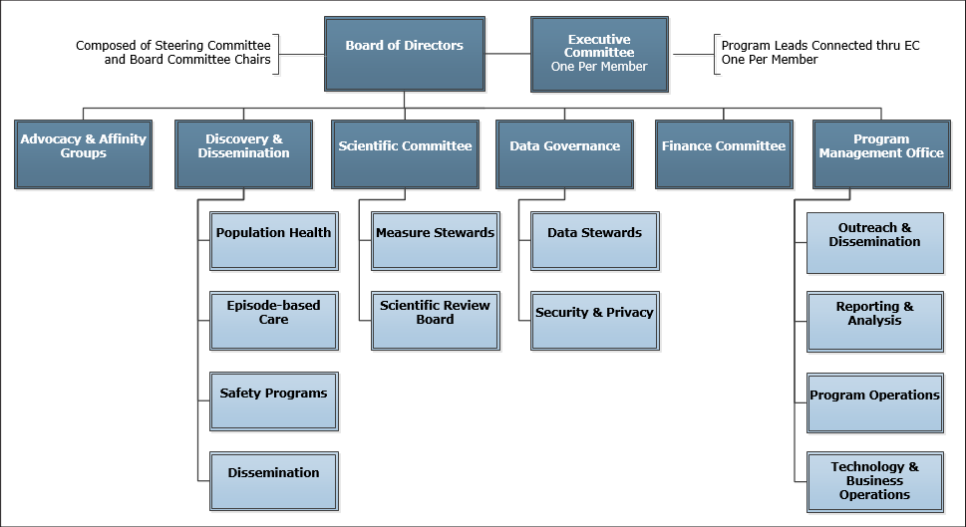
HVHC Governance Structure.

The Steering Committee remains in place as authorized in the MCA. A non-traditional board structure with five committees and an overarching chair provides the functional working core that includes: Finance, Discovery and Dissemination, Data Governance, Scientific, and Advocacy. The latter four Board committees include an elected Chair and Vice-Chair, who are required to be members of the Executive Committee to be Board eligible. Each of these committees has focused Program areas supporting collaborative work that are led by staff from member organizations. Both Board committees and Programs are supported by staff from the PMO. The CEO of the PMO reports to the Chair of the Board. Figure 1 depicts the organizational chart for oversight of HVHC work.

The HVHC Board is accountable for establishing and achieving the vision and mission of the High Value Healthcare Collaborative. The Executive Committee represents the collective voice of the members and make decisions on behalf of their individual member institutions. Member Program Leads are responsible for resourcing and overseeing HVHC projects in which their institutions are participating.

The Discovery & Dissemination Committee is the unifying structure for care improvement and research efforts; Collaborative home for incubation of innovative ideas; and this structure is intended to provide support for our transformational work. The intent of the Discovery and Dissemination Subcommittee is to manage scarce resources, support shared learning, and align Collaborative-led efforts resourced by the PMO with HVHC priorities.

The Scientific Committee optimizes member value by facilitating, supporting, and ensuring the scientific integrity of HVHC work products including measure stewardship. The Scientific Committee has two subcommittees: Scientific Review Board (SRB) and Measure Steward Subcommittee. The SRB provides technical oversight for proposed HVHC projects (research, improvement, and advocacy commentary) and consultation on the following: scientific validity and integrity of methods; ability to contribute to current gaps in literature and knowledge; and relevance to HVHC mission for methods. The Measure Steward Subcommittee optimizes member value by overseeing the accuracy of measures used to support the HVHC work products.

The Data Governance Committee optimizes the value of data for HVHC Members and facilitates secure and appropriate access to data in support of collaborative-led and member-initiated projects. The Data Governance Committee has two subcommittees. The Data Stewards Subcommittee develops data mobilization strategies, specs, and access rules based on the needs of data-oriented HVHC projects. The Security & Privacy Subcommittee provides oversight for policies, procedures, audit response, and incident response related to all data transmitted to/from and housed by the PMO on behalf of the Collaborative.

The Advocacy & Affinity Groups Committee (AAGC) provides a structure to allow the HVHC to advance specific creative ideas to stimulate legislation, regulation and sustainable payment to support healthcare models that foster value. Affinity Groups that are created in the HVHC by approval of the HVHC Executive Committee provide specific interactions with HVHC member organizations that help support the HVHC Mission as well as individual member organization needs.

The Finance Committee is responsible for recommending financial policies, goals, and budgets that support the mission, values, and strategic goals of HVHC. The committee also reviews HVHC’s financial performance against goals and proposes changes in fees and programs to the board.

## HVHC Data Trust

A critical component to the HVHC approach is the data infrastructure, analytics, and tools. Using a highly secure data infrastructure, the PMO receives, cleanses, standardizes, and analyzes clinical and cost data from members and other sources, including data for almost 40M Medicare beneficiaries. HVHC members use comparative analytics to support pilot projects, research studies, and benchmarking. HVHC tools provide members with web-based querying capabilities, comparative reporting to monitor progress and impact of HVHC projects, and access to specific data marts for IRB-approved research.

HVHC has put in place comprehensive policies and procedures under the CMS requirement to adhere to standards of NIST Special Publication 800-53 “Recommended Security Controls for Federal Information Systems and Organizations” (also known as NIST-853). In January 2013, HVHC and its data center received confirmation from a third-party data security consulting organization that the infrastructure at Dartmouth College used to support the processing and storage of externally-provided data is aligned with NIST 800-53 requirements.

Central to the HVHC mission is the use of different types of data to identify improvement areas for its Members. The HVHC PMO currently maintains four types of data from sources on secure servers:

**Table:** 

Data Type	Data Source

Administrative (e.g., discharge data, birth records)	Member
Clinical (e.g., procedure details, lab results)	Member
Patient Generated Health Data (e.g., PHQ-9, PROMIS)	Member/Vendor
Medicare Claims (e.g., inpatient, outpatient)	CMS

The PMO also regularly processes reference data from various sources (e.g., Medicare Provider of Service list, American Hospital Association) to support analytic and data operations activities. Members also send patient identifiers solely for the purpose of linking data to other data sources. Once the data are linked, the patient identifiers are stored in a separate and secure location. Once a project is approved by Dartmouth’s Institutional Review Board (and appropriate data use agreements are in place if using outside data sources such as Medicare claims data), Limited Data Sets (LDSs) are provided to authorized users for analysis, de-identified except for dates and ZIP codes.

HVHC data sources are received in several ways:

- Member-submitted data: submitted via SFTP upload. Upon file receipt, files are automatically pushed to a secure, hidden environment for PMO Data Operations staff to retrieve; Member Data Operations personnel cannot retrieve or view files post-upload (noted by the star in Figure 1). The PMO Data Operations staff loads data to the HVHC Data Trust from this hidden environment.
- CMS-provided claims data: The PMO Data Operations staff manually loads data to the HVHC Data Trust from physical media (or other method specified by CMS). Any physical media mailed to the PMO are stored in a secure physical environment in PMO offices.
- Vendor-provided Member survey data: Survey response data are downloaded by the PMO through a secure login to third-party online platform, PMO Data Operations staff then loads data to the HVHC Data Trust.

A more complete description of the member-submitted data process is provided by Priest et al. [4].

## HVHC Discovery and Dissemination Process

**Figure 2:**
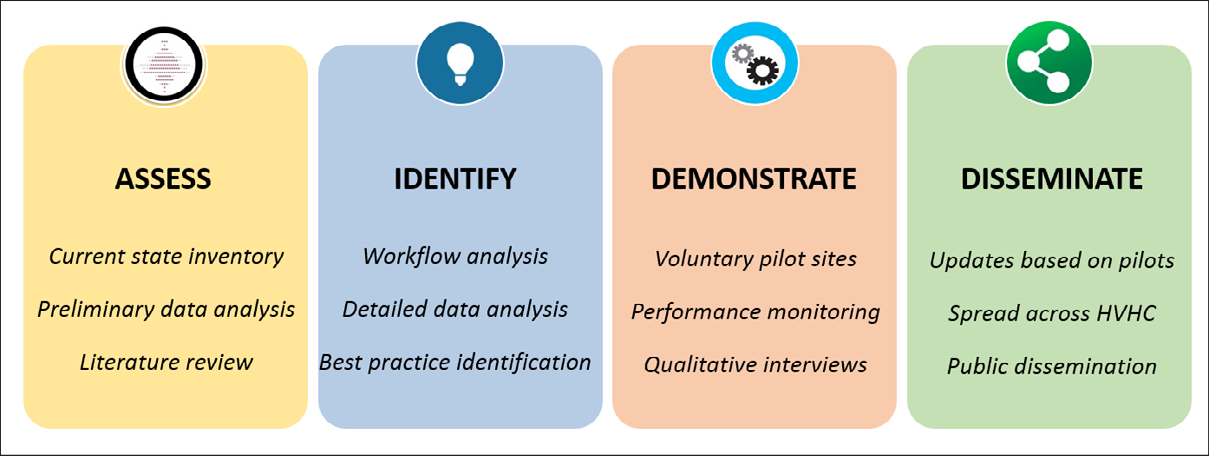
HVHC Discovery & Dissemination Process.

Multi-site, multi-disciplinary teams work together to assess high-cost, high-variation health conditions and treatments, identify promising practice models of care aligned with better value-based measures, demonstrate improvement in value via multi-site pilot projects, and then disseminate proven best practice models across HVHC and to the public. This sequential learning process is overseen by a Scientific Review Committee. Figure 2 provides a graphical depiction of the discovery and dissemination process.

A key success factor to this discovery and dissemination process is transparency of performance measures and shared learning. To facilitate this, HVHC built two Web-based portals for Member access: the HVHC Resource Portal and the Vantage Data Reporting Portal. One example of completed, comparative work is reported by Tomek et al. [5].

## Work Featured in This Special Issue

The HVHC was awarded a grant from the John and Laura Arnold Foundation to develop a generalizable model for dissemination and implementation. Implementation of the 3-hour sepsis bundle was used as a prototypic, complex intervention with an in-depth mixed methods evaluation across 16 member sites. The first four articles in this issue describe, in detail, various data and methodological challenges encountered together with strategies for overcoming these (see Knowlton et al., von Recklinghausen et al., Welch et al., and Taenzer et al.).

Next, we illustrate how the Data Trust can support emerging questions relevant to member organizations. The paper by Albritton et al., explores the impact of observation stays on readmission rates. Knighton et al., explore the use of an area-based measure for health literacy to assess risk in disadvantaged populations.

Two final papers illustrate the importance of fundamental data sources needed to support advanced data science. James et al., describe an approach to capturing computable data as part of the care delivery process in the electronic health record. Lastly, Cohen et al., describe how the Agency for Healthcare Research and Quality (AHRQ) Centers of Excellence have leveraged diverse data sources in describing U.S. delivery systems—a crucial but previously unavailable standard for comparative analytics (note: TDI with HVHC is one of these awardees).
